# NOTCH1 signaling promotes protein stability of HER3 through the AKT pathway in squamous cell carcinoma of head and neck

**DOI:** 10.1038/s41389-021-00348-5

**Published:** 2021-08-31

**Authors:** Yi-Ping Wang, I-Ju Liu, Kai-Chi Chen, Han-Chung Wu

**Affiliations:** 1 Faculty of Dentistry, School of Dentistry, National Taiwan University, Taipei, Taiwan; 2 Institute of Cellular and Organismic Biology, Academia Sinica, Taipei, Taiwan; 3 Department of Dentistry, National Taiwan University Hospital, Taipei, Taiwan; 4 Graduate Institute of Oral Biology, School of Dentistry, National Taiwan University, Taipei, Taiwan

**Keywords:** Head and neck cancer, Cell signalling

## Abstract

Epidermal growth factor receptor (EGFR) remains the sole druggable molecular target other than the PD1/PD-L1 pathway with meaningful clinical benefit in squamous cell carcinoma of head and neck (SCCHN). Human epidermal growth factor receptor 3 (HER3) confers the resistance to EGFR-targeted treatment in SCCHN. Thus, it is essential to determine the distribution and regulatory mechanisms of HER3 in SCCHN. We explored the prevalence of HER3 expression and its distribution within SCCHN by immunohistochemical staining and clinicopathological correlations were analyzed. The regulatory mechanism of HER3 expression was then dissected in vitro, using RT-PCR, Western blotting, and immunoprecipitation in a set of SCCHN cell lines. Subsequent in vivo validation in the murine model was also performed. We found that concomitant high expression of HER3 and its ligand NRG1 in SCCHN is associated with the increased presence of regional lymphatic metastasis and the majority of HER3 is located on the differentiated tumor cells. Further investigation revealed that HER3 is under positive control of NOTCH1 through transcriptional activation and inhibition of protein degradation through the polyubiquitination machinery via AKT pathway and USP8 deubiquitinating enzyme. In addition, loss of function of NOTCH1 suppresses HER3 expression through increased phosphorylation of serine 473 of AKT in SCCHN cells, and promotes the aggressiveness of the tumor cells. These data indicated that the level of HER3 is regulated by NOTCH1 in SCCHN both transcriptionally and post-translationally, and NOTCH1 is in a higher hierarchy in the regulatory system of the AKT pathway. Since NOTCH1 is inactivated in approximately 10% of SCCHN cases and this aberration strongly impacts the AKT pathway and diminishes HER3, exclusion of patients with NOTCH1-inactivated SCCHN may be beneficial for future clinical trials of HER3-targeting antibodies.

## Introduction

Squamous cell carcinoma of the head and neck (SCCHN) poses a grave threat to public health in South-Central Asia, Melanesia, and Central and Eastern Europe, with 830,000 new cases and 430,000 SCCHN-related deaths reported worldwide annually [1]. This type of cancer comprises two major subtypes, namely, the conventional type SCCHNs and the HPV-associated oropharyngeal SCC [2]. The former usually arises from potentially malignant disorders within the mucosa lining the oral cavity, oropharynx, hypopharynx, and larynx [3], and this entity shares a similar biological behavior and hence a similar staging system [4]. Regional lymph node involvement is a common feature of patients with advanced-stage SCCHN (stages III to IVB), which comprise about two-thirds of total SCCHN cases [5]. Despite recent advances in the fields of surgery and oncology, the failure rate of locoregional control is estimated to be up to 60%, and the risk of distant metastasis is up to 30% [6, 7]. In addition, the overall survival of SCCHN patients has only increased by 5% over the last two decades [8].

Intensive research on the oncophysiology of SCCHN has been conducted, however, epidermal growth factor receptor (EGFR) remains the sole druggable molecular target with meaningful benefit, other than immune-related pathways [2, 9–12]. Cetuximab, a chimeric immunoglobulin G1 (IgG1) antibody against EGFR, is approved for use in conjunction with radiotherapy in locoregionally advanced SCCHN or as a single regimen in platinum-refractory, recurrent/metastatic SCCHN [13, 14]. In recent studies, mounting evidence suggests that compensatory HER3 signaling and subsequent phosphatidylinositol-3-kinase (PI3K)/AKT activation plays a major role in resistance to EGFR-targeted treatments in SCCHN [15, 16]. Thus, several HER3-targeting biological drugs are under development and have entered clinical trials. HER3 exhibits a weak kinase activity compared to other HER family members [17], however, the cytoplasmic domain of HER3 possesses six docking sites for the p85 regulatory subunit of PI3K. Upon binding of its ligand, neuregulin 1 (NRG1), HER3 serves as an efficient phosphotyrosine scaffold to other HER family members and causes potent activation of downstream AKT signaling. Nevertheless, the regulatory mechanisms underlying HER3 expression and turnover have not been fully elucidated.

In this study, we evaluated the levels of HER3 and NRG1 in SCCHN and analyzed the associations with clinical outcomes. Concomitant high expression of these two proteins in SCCHN was associated with increased regional lymphatic metastasis. In addition, the majority of HER3 was found to be located on differentiated tumor cells in the center of infiltrative tumor nests. The differentiation of keratinocytes in the stratified squamous epithelium is regulated by several master molecules, including p63 and NOTCH1. As the differentiation progresses, the keratinocytes gradually lose expression of p63, and NOTCH1 becomes a major determinant of cell fate [18, 19]. We demonstrated that HER3 is under the control of NOTCH1, which stimulates HER3 transcriptional activation and inhibits its protein degradation. Interestingly, loss of function of NOTCH1 suppressed the expression of HER3 but boosted the phosphorylation of AKT serine 473 (S473) in SCCHN cells, suggesting that NOTCH1 affects the PI3K/AKT pathway more profoundly and independently of its effects on HER3. Based on these findings, it may be beneficial to exclude patients with dysfunctional NOTCH1 from clinical trials on HER3-targeting therapeutics, and expression of NRG1 may serve as a valuable biomarker for enrollment in such trials.

## Materials and methods

### Clinical specimens and clinicopathological staging

The protocols and procedures in this study conformed to all relevant ethical and institutional guidelines and were conducted after the acquisition of informed consent from all subjects and approval by the Institutional Review Board of National Taiwan University Hospital. Since the first choice of treatment in laryngeal and pharyngeal SCC has been shifted to definitive chemoradiotherapy to achieve organ preservation [8], the quantity of the specimens from those two regions has been dwindled and the oral cavity is the only anatomical location that offers ample surgical specimen of head and neck squamous cell carcinoma. We retrieved one hundred FFPE tissue blocks of oral squamous cell carcinoma (OSCC) patients from the archive at the Department of Oral Pathology in the years 2006–2011 (NTUH IRB, No. 201412066RINA and No. 201901040RIND) to ensure adequate statistical power. The preset enrolled criteria were primary oral squamous cell carcinoma without previous treatment (such as inductive chemotherapy and radiation therapy) and the age of the patient was required to be older than 20 years. The exclusion criteria was set as recurrent cancers, positive history of previous chemo/radiotherapy, and tissue size smaller than 0.7 × 0.5 cm. The diagnosis was confirmed after a review of hematoxylin and eosin-stained (H&E) tissue sections by two qualified oral pathologists. The TNM staging at the time of diagnosis for every case was re-evaluated according to the 8^th^ edition of staging criteria from the American Joint Committee on Cancer. Complete records of follow-up were available in 87 out of 100 cases enrolled. The recurrence of the tumor was defined as an SCC found less than 2 cm away from and within 3 years of the previous primary tumor [20]. Areas that were 1 cm away from the peritumoral epithelial dysplasia and devoid of an underlying inflammatory cell infiltrate were considered as normal mucosa (*n* = 11). The clinical characteristics of the enrolled cohort were summarized in Supplementary Table S1.

### Immunohistochemical (IHC) staining of HER3 and NRG1

Tissue sections from enrolled cases were cut to the 4 μm thickness and stained by immunohistochemistry. Routine immunohistochemical staining was performed with monoclonal primary antibodies against HER3 and NRG1 (details in Supplementary Table S2), using a protocol described previously [21]. Tissue sections incubated with normal mouse IgG (NMIgG) instead of primary antibody served as negative controls. Perceptible and convincing granular membrane staining, either partially or completely encircling the cells, was regarded as positive. All histopathological images were captured with an Olympus BX53 microscope and DP2-BSW image acquisition software.

### Cell lines and culture conditions

FaDu (HPV-negative oropharyngeal squamous cell carcinoma) and Cal 27 (OSCC) cell lines were purchased from the Bioresource Collection and Research Center (BCRC, Hsinchu, Taiwan). Both cell lines were maintained in medium (MEM for FaDu cells and DMEM for Cal 27 cells) supplemented with 10% fetal bovine serum (FBS; Gibco, USA) and 100 μg/ml Penicillin/Streptomycin (P/S; Gibco, USA) in a humidified incubator at 37 °C with 5% CO_2_. The authenticity of cell lines was confirmed by short tandem repeat analysis and negativity of mycoplasma contamination was routinely checked through PCR every week.

### Immunoprecipitation of HER3 and polyubiquitin assays

Cells were transfected with a pEF1α-HER3 plasmid for 24 h (hs), and cells were treated with MG132 (25 μM) for 5 h. Subsequently, cells were mixed with lysis buffer (50 mM Tris-HCl, pH 7.4, 150 mM NaCl, 1% NP-40) supplemented with protease inhibitors (Roche, Switzerland). Equal amounts of total proteins (500 μg) were incubated with the anti-HER3 antibody at a dilution of 1:250 at 4 °C for 8 h. Then, proteins were incubated with 10 μl Dynabeads ^TM^ Protein G (Invitrogen, USA) at 4 °C for 2 h to pull down the antibody-bound proteins. The mixtures were washed and boiled in SDS sample buffer (50 mM Tris-HCl, pH 6.8, 2% SDS, 0.1% bromophenol blue, 10% glycerol, 12.5% β-mercaptoethanol). The pulled-down proteins were analyzed for ubiquitinylation by Western blotting.

### Phospho-RTK array assay

The profile of phosphorylated-receptor tyrosine kinases (RTKs) was analyzed using a Human Phospho-RTK Array Kit (R&D Systems, USA), according to the manufacturer’s instructions. The intensity score of each array spot was measured with a UVP BioSpectrum image system. Phosphorylated RTK protein levels were compared between shNOTCH1 SCCHN cells and the corresponding control cells (pLKO groups).

### Subcutaneous xenograft model

Female non-obese diabetic/severe combined immunodeficiency (NOD/SCID) mice were obtained from the Jackson Laboratory (Bar Harbor, Maine, USA) and were bred in the core facility of the ICOB at Academia Sinica, Taiwan. As per the regulation of Institutional Animal Care and Use Committee of Academia Sinica, Taiwan (IACUC No. 20-05-1468), mice of 4–6 weeks old were injected subcutaneously with 2 × 10^6^ NOTCH1-wide type (WT) and NOTCH1-knockout (KO) Cal 27 cells in each side of the dorsolateral flank (*n* = 8). No randomization of mice was performed in this experiment. Mice that did not grow tumors will be excluded from the experiment, however, all inoculated mice in this experiment developed tumors as expected. The tumor volumes were calculated using the following formula: tumor volume = length × (width)^2^ × 0.52. The data are presented as the mean ± standard error of the mean.

### Statistics

The association between clinicopathological parameters and expression status of HER3 and NRG1 in clinical specimens was analyzed by Fisher’s exact test and Pearson’s chi-squared test. All in vitro comparisons were performed with three technical replicates and three independent biological replicates (*n* = 3). Analyses of in vitro experiments were performed using two-tailed ANOVA. *P*-values less than 0.05 were considered statistically significant for all tests. All statistical analyses were performed using SPSS (SPSS Inc., USA).

## Results

### Concomitant high expression of HER3 and NRG1 is associated with nodal metastasis in SCCHN and HER3 is regulated by NOTCH1

**Fig. 1:**
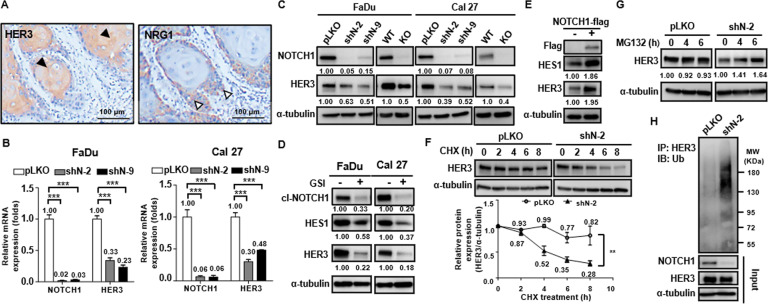
HER3 is preferentially expressed in differentiated tumor cells and inhibition of NOTCH1 suppresses HER3 mRNA and destabilizes HER3 protein in SCCHN. **A** The majority of HER3-positive cells were differentiated cells in the center of the infiltrating tumor nests (black arrowhead, membranous stain of HER3), while the signal for NRG1 was primarily found in basaloid cells at the periphery of the tumor nests (white arrowhead, cytoplasmic stain of NRG1). **B** The mRNA level of HER3 was drastically reduced in FaDu and Cal 27 cells after NOTCH1 silencing with different lentivirus-expressed shRNAs, when compared to control shRNA (pLKO, as the baseline of normalization). Column: the average relative expression, compared to the pLKO group ± standard error (*n* = 3). ***p < 0.001 by two-way ANOVA. **C** The protein level of HER3 was also greatly reduced in FaDu and Cal 27 cells after NOTCH1 silencing with different shRNAs or knockout with a CRISPR-Cas9 system. **D** The expression of HER3 was significantly decreased after NOTCH1 signaling was inhibited with the treatment of a γ-secretase inhibitor (GSI) for 24 h in SCCHN cells (FaDu, 50 μM; Cal 27, 20 μM). cl-NOTCH1, cleaved NOTCH1; HES1, a known downstream component of NOTCH1 pathway that serves as an indicator of NOTCH1 signaling. **E** Conversely, overexpression of NOTCH1 protein efficiently increased the expression of HES1 and HER3. **F** The protein stability assay revealed a steep decrease of HER3 protein and a lower residual protein quantity in NOTCH1-knockdown FaDu cells compared to controls (28% vs. 82% at 8 h) after inhibition of translational activity by cycloheximide (30 μg/μl). Dot: the average relative expression normalized to the original level ± standard error (*n* = 3). **p < 0.01 by two-way ANOVA. **G** The downregulation of HER3 protein in NOTCH1-knockdown cells could be reversed by the addition of the proteasome inhibitor MG-132 (25 μM). **H** Immunoprecipitation assay showed enhanced polyubiquitination of HER3 protein in NOTCH1-knockdown cells compared to control cells (pLKO).

To investigate the clinical significance of the HER3 pathway in SCCHN, we first evaluated the expression status of HER3 and its ligand NRG1 in 100 cases of OSCC, which is the 4th leading cancer type in the male population in Taiwan, through immunohistochemical staining. Positive membranous stains of HER3 and NRG1 were discerned in 95 cases. The mean and standard deviation of the percentage of positive tumor cells was 72.93 ± 31.68% for HER3 and 81.65 ± 21.84% for NRG1, respectively. On the other hand, the percentage of the keratinoyctes positive for HER3 was significantly lower in all eleven samples of the normal epithelium (51.51 ± 5.09%, *p* = 0.0012) and no signal of NRG1 was seen in any normal keratinocytes (Supplementary Fig. S1A). Analysis of the TCGA dataset of SCCHN on UALCAN platform also revealed that the mRNA transcript number of NRG1 was significantly higher in primary SCCHN than the normal counterparts (Supplementary Fig. S1B). We found no significant correlation between these two markers and parameters in the TNM system when evaluated in univariate analysis (data not shown). However, simultaneous high expression of HER3 and NRG1 within the tumor (percentages of positive neoplastic cells were more than the mean value of corresponding markers) was significantly associated with the presence of metastasis and high cancer staging, and the presence of local recurrence (Table 1). Concomitant high expression of these two markers failed to demonstrate a significant correlation of the presence of extracapsular spread at the time of diagnosis (Table 1), and no significant difference of 5-year overall survival was discerned between the concomitant NRG1 and HER3 high-expressing subset and the rest of the enrolled cases (Supplementary Fig. S2). In addition, we observed that the majority of the HER3-positive cells were the differentiated cells in the center of the infiltrating tumor nests, and the NRG1-positive cells were primarily the basaloid cells at the periphery of the cell nests (Fig. 1A). This spatial discrepancy was discerned in 66% of the enrolled cases. This phenomenon drives us to explore if the differentiation axis of the squamous epithelium takes a part in the regulation of HER3. Given the fact that NOTCH1 is a master regulator in the terminal differentiation of the squamous epithelium and HER3 was mostly observed in the more differentiated tumor cells, we evaluated the contribution of NOTCH1 to the expression of HER3. Immunohistochemical staining of NOTCH1 was performed in our cohort of OSCC. It revealed that the expression of NOTCH1 protein in the cases without nodal involvement was approximately two-fold higher than those cases with nodal diseases, and downregulation of NOTCH1 was closely associated with nodal involvement (*p* = 0.0865, Supplementary Fig. S3B). Colocalization of NOTCH1 and HER3 on the centrally-located differentiated neoplastic cells within the infiltrative tumor nests was also discerned in 51% of cases of our enrolled cohort (Supplementary Fig. S4). However, this finding did not confer any statistically significant difference in regard to clinicopathological features (data not shown). In vitro studies showed that the level of HER3 mRNA was drastically reduced after the knockdown of NOTCH1 through various clones of shRNA in both FaDu and Cal 27 cell lines (Fig. 1B). Efficient suppression of HER3 protein was also evident after knockdown and knockout of NOTCH1 with different constructs of shRNA and CRISPR-Cas9 system in SCCHN cells (Fig. 1C). Furthermore, we found that the expression of HER3 was dose-dependently reduced by treatment of NRG1 in increasing concentrations in NOTCH1-knockout FaDu cells and Cal 27 cells (Supplementary Fig. S5), and this reduction was also present in the NOTCH1-expressing counterparts when exposed to NRG1 in high concentration (40 ng/ml). Since the activation of the canonical NOTCH1 pathway requires protein cleavage by γ-secretase to release the NOTCH intracellular domain (NICD) to trigger the downstream transcription, we further assayed the impact of functional disability of NOTCH1 upon the regulation of HER3. After adding an inhibitor of γ-secretase, the protein levels of HES1 (a known downstream component of NOTCH1 pathway, as a control) and HER3 were significantly reduced in SCCHN cells (Fig. 1D). Conversely, overexpression of NOTCH1 protein efficiently increased the expression of HES1 and HER3 (Fig. 1E). Taken together, it suggests that NOTCH1 positively regulates HER3 on both mRNA and protein levels in SCCHN.

**Table 1:** Clinicopathological correlation of concomitant high expression of HER3 and NRG1 and OSCC outcome.

		Concomitant high expression of HER3 and NRG1		Pearson *p*-value
Parameter		(−)	(+)	
***T***				
	T1 + T2	48 (72.73%)	18 (52.94%)	0.079
	T3 + T4	18 (27.27%)	16 (47.06%)	
***N***				
	N0	50 (75.76%)	17 (50.00%)	0.018
	N1 + N2 + N3	16 (24.24%)	17 (50.00%)	
***Stage***				
	I + II	49 (74.24%)	17 (50.00%)	0.028
	III + IV	17 (25.76%)	17 (50.00%)	
***Recurrence***^a^				
	Negative	46 (82.14%)	15 (48.39%)	0.002
	Positive	10 (17.86%)	16 (51.61%)	
***ENE***^b^				
	Negative	12 (75.00%)	14 (82.35%)	1.000
	Positive	4 (25.00%)	3 (17.65%)	

^a^Complete records of follow-up were available in 87 out of 100 cases enrolled.

^b^*ENE,* extranonal extension.

### Inhibition of NOTCH1 compromises HER3 protein stability

The expression level of protein is determined by various mechanisms, including transcriptional activation and proteosomal degradation. Next, we examined the difference of protein stability of HER3 with and without the suppressed function of NOTCH1 in SCCHN cells. The protein level of HER3 was prematurely decreased to 52% of the original level at 4 h after inhibition of translational activity through cycloheximide in NOTCH1-knockdown SCCHN cells, and it further dropped to 28% at post-treatment 8 h. In contrast, the protein level of HER3 remained as 99% and 82% of the original level in the control counterpart at the corresponding time points, respectively (Fig. 1F). These findings suggested NOTCH1 affects not only the transcriptional regulation but also the protein stability of HER3 through manipulating the proteosomal degradation. To test our hypothesis, we added the proteosomal inhibitor MG132 into the culture media of NOTCH1-knockdown SCCHN cells and the control counterpart. The downregulation of HER3 protein in NOTCH1-knockdown cells could be reversed through the addition of the proteasome inhibitor MG132 (Fig. 1G). Since the polyubiquitin tag serves as the beacon for the 26 S proteasome and is a pivotal marker in protein degradation, we next investigated if NOTCH1 influences the polyubiquitination of the HER3 protein. After overexpression of HER3 in NOTCH1-knockdown SCCHN cells and the control counterpart, immunoprecipitation of HER3 was performed and the presence of ubiquitin on the protein was probed with Western blotting. Enhanced polyubiquitination was discerned in NOTCH1-knockdown cells compared to the control cells (Fig. 1H). In summary, these data indicated NOTCH1 not only participates in the transcription of HER3 but also enhances the protein stability of HER3 through inhibition of polyubiquitination of the HER3 protein.

### Loss of NOTCH1 function leads to activation of AKT signaling

**Fig. 2:**
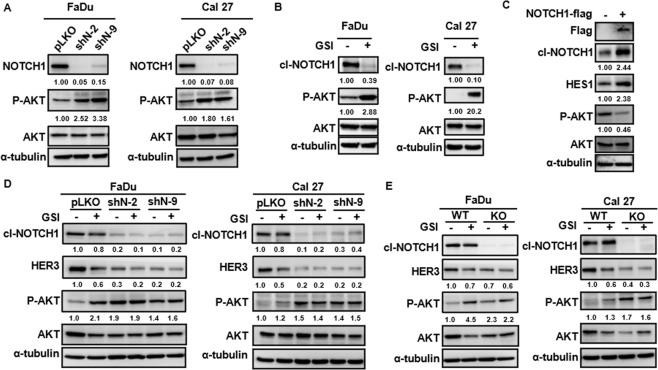
NOTCH1 negatively regulates AKT in SCCHN cells. **A** Increased phosphorylation of AKT was observed in FaDu and Cal 27 cells after NOTCH1-knockdown with different shRNA constructs. **B** Phosphorylation of AKT was also enhanced by treatment with GSI in FaDu and Cal 27 cells. **C** Overexpression of NOTCH1 led to decreased phosphorylation of AKT in FaDu cells. **D** The addition of GSI to NOTCH1-knockdown FaDu and Cal 27 cells did not further boost the phosphorylation of AKT. **E** Phosphorylation of AKT remained the same in NOTCH1-knockout FaDu and Cal 27 cells after treatment with GSI.

In order to discover the underlying mechanism of NOTCH1-inhibited polyubiquitination of HER3, we explored the change of signaling landscape after the knockdown of NOTCH1 in SCCHN cells. The analysis of phosphorylated-receptor tyrosine kinase array revealed that the only alteration of the major signaling pathway in both cell lines with a meaningful fold-change after knockdown of NOTCH1 was the augmentation of phosphorylation of serine 473 of AKT (6.71 folds in FaDu cells and 5.18 folds in Cal 27 cells, Supplementary Fig. S6). This finding was unexpected since HER3 is known as an activator of AKT and downregulation of HER3 through inhibition of NOTCH1 was supposed to result in suppression of the AKT pathway. This finding suggested that NOTCH1 is in a higher hierarchy in the AKT axis than HER3, since the effect of NOTCH1 upon the AKT pathway overrides that of HER3. In order to verify if this is a bystander effect caused by shRNA, we examined the phosphorylation of AKT in SCCHN after manipulation of the function of NOTCH1 with various methods. Increased phosphorylation of AKT was present when the NOTCH1 was knockdown with different constructs of shRNA (Fig. 2A) and when the function of NOTCH1 was inhibited by a γ-secretase inhibitor (Fig. 2B), while overexpression of NOTCH1 led to decreased phosphorylation of AKT (Fig. 2C). Furthermore, the addition of γ-secretase inhibitor to NOTCH1-knockdown and NOTCH1-knockout SCCHN cells did not boost the phosphorylation of AKT, indicating the AKT-stimulating effect was only associated with NOTCH1 but not other substrates of γ-secretase (Fig. 2D and Fig. 2E, respectively). To seek out the possible underlying mechanism of AKT activation after the loss of function of NOTCH1, we examined the impact on the phosphorylation landscape of EGFR after knockdown of NOTCH1 in FaDu and Cal 27 cells. Although augmentation of phosphorylation of AKT was consistently observed, the phosphorylation signatures of EGFR were generally decreased after knockdown of NOTCH1 in FaDu cells, while phosphorylation of EGFR at all tested loci were upregulated by the shN-9 clone in Cal 27 cells. (Supplementary Fig. S7). A more detailed multi-omic elucidation of these two cell lines and translation into clinical specimens is needed in a future study to unravel the underlying cause of this discrepancy.

### Activation of AKT contributes to destabilization of HER3 through negative regulation of USP8

**Fig. 3:**
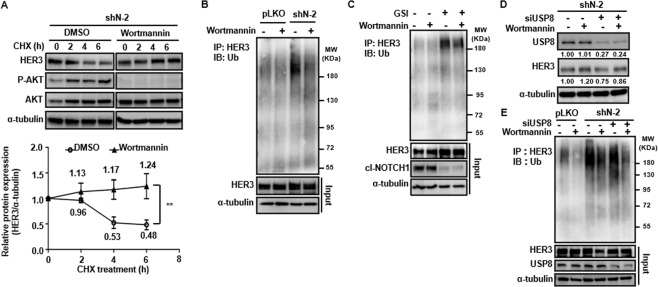
NOTCH1 downregulation promotes degradation of HER3 protein via suppression of deubiquitinating enzyme USP8 through activated AKT signaling. **A** The protein level of HER3 steadily increased over 6 h following treatment with wortmannin (10 μM) in HER3-recapitulated, NOTCH1-knockdown SCCHN FaDu cells. In contrast, the HER3 protein level was less than 50% of the original level in the DMSO-treated control group. Dot: average relative expression normalized to the original levels ± standard error (*n* = 3). **p < 0.01 by two-way ANOVA. **B** Immunoprecipitation experiments also showed wortmannin lowered HER3 polyubiquitination in both control FaDu cells (two lanes on the left) and NOTCH1-knockdown cells (two lanes on the right). Polyubiquitination of HER3 was more prominent in NOTCH1-knockdown cells than in control cells. **C** Similar results were found when the NOTCH1 function was compromised by GSI. **D** When USP8 was stimulated by wortmannin, the protein level of HER3 in NOTCH1-knockdown FaDu cells increased (two lanes on the left), while HER3 protein was reduced after the introduction of siRNA against USP8 (two lanes on the right). In addition, treatment with wortmannin partially rescued the expression of HER3. **E** Ubiquitination of HER3 was decreased in both control and NOTCH1-knockdown SCCHN cells after treatment with wortmannin, which resulted in enhancement of USP8. This suppression could be partially reversed by the siRNA knockdown of USP8.

**Fig. 4:**
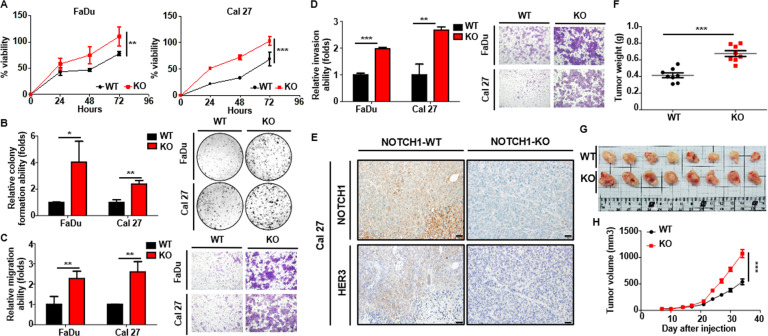
Loss of function of NOTCH1 down-regulates HER3 expression and augments tumor aggressiveness in SCCHN. Functional assays were performed and a comparison between the NOTCH1-wild type (WT) and NOTCH1-knockout (KO) SCCHN cells. Knockout of NOTCH1 significantly promotes the cellular viability (**A**), the capability of colony formation (**B**), migration (**C**), and invasion (**D**) in both FaDu and Cal 27 cells. Data are presented as the mean ± SD. *p < 0.05; **p < 0.01 and ***p < 0.001 (**E**) Immunohistochemical staining revealed that the expression of HER3 was positively correlated with NOTCH1 in the xenografted Cal 27 cells in the murine subcutaneous xenograft model. The tumor volumes (**F**, **G**) of the NOTCH1-knockout tumors are significantly increased compared to those of the NOTCH1-wild type tumors. Scale bar, 50 μm. Data are presented as the mean ± SEM. ***p < 0.001.

**Fig. 5:**
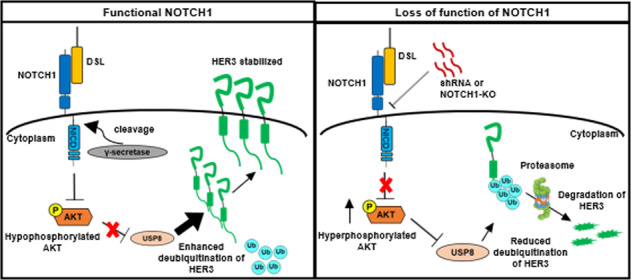
Graphical abstract. Schematic illustration shows the working model of NOTCH1 regulation of HER3 protein. Functional NOTCH1 signaling suppresses the phosphorylation of AKT serine 473, which inactivates the deubiquitinating enzyme USP8. Activated USP8 then deceases the polyubiquitination of HER3, preventing proteasomal degradation of HER3 protein (left). When loss of function of NOTCH1 occurs, phosphorylation of AKT is increased, and the activity of USP8 is subsequently down-regulated. Once the deubiquitination is suppressed, the poly-ubiquitinated HER3 protein is subject to proteasomal degradation (right).

Next, we investigated whether activation of AKT is a pivotal component in NOTCH1-inhibited polyubiquitination of HER3. The protein level of re-expressed HER3 kept increasing in the 6 h under treatment of wortmannin (10 μM) in NOTCH1-knockdown SCCHN cells, in contrast, the protein of HER3 dwindled to less than 50% of the original level in the DMSO-treated control group (Fig. 3A). Immunoprecipitation of HER3 also showed that wortmannin lowered the polyubiquitination of HER3 in both control SCCHN cells and the corresponding NOTCH1-knockdown counterpart, and the polyubiquitination of HER3 was more prominent in NOTCH1-knockdown cells than that discerned in the control cells (Fig. 3B). Similar results were also present when the function of NOTCH1 was compromised by γ-secretase inhibitor in SCCHN cells (Fig. 3C). These findings confirmed that activation of the PI3K/AKT pathway enhances polyubiquitination of HER3, and downregulation of NOTCH1 further boosts the degradative signal of HER3. Polyubiquitination is a multistep process and is controlled by a set of enzymes, typically composed of activating enzymes, conjugating enzymes, and ligase. We examined the mRNA of two E3 ligases (NEDD4 and NRDP1) in SCCHN cells after knockdown of NOTCH1 and no significant difference was noted compared to that in the control counterpart (data not shown). Then, we examined components of the polyubiquitination process that are regulated by the AKT axis. Ubiquitin-specific peptidase 8 (USP8) is a deubiquitinating enzyme under negative regulation of the PI3K/AKT pathway. When the function of USP8 was enhanced by wortmannin, the protein level of HER3 in NOTCH1-knockdown SCCHN cells increased (Fig. 3D, two lanes on the left). After the introduction of siRNA against USP8, HER3 protein were reduced due to suppressed deubiquitination, and a supplement of wortmannin partially rescued the expression level of HER3 (Fig. 3D, two lanes on the right). Immunoprecipitation and subsequent immunoblotting also confirmed that the ubiquitination of HER3 decreased in both control and NOTCH1-knockdown SCCHN cells after treatment with wortmannin, which resulted in enhancement of USP8, and this suppression can be partially reversed by inhibition of USP8 through siRNA (Fig. 3E). However, loss of NOTCH1 exerts a more profound impact on the biological behavior of SCCHN cells than that caused by decreased HER3. Knockout and knockdown of NOTCH1 in FaDu and Cal 27 cells enhanced the cellular viability and the capabilities of colony formation, migration, and invasion, albeit the effects of shRNA clone shN-9 were less prominent in migration assay of Cal 27 cells and invasion assays in FaDu cells (Fig. 4A, D and Supplementary Fig. S8). Murine in vivo subcutaneous xenograft model also demonstrated the expression of HER3 was positively correlated with NOTCH1 in the xenografted Cal 27 cells (Fig. 4E), and volumes of the tumors from NOTCH1-knockout Cal 27 cells were significantly larger than those from the NOTCH1-wild type counterpart (Fig. 4F, G). Phosphorylation of AKT was also enhanced in NOTCH1-knockout tumors than that in the NOTCH1-wild type lesions (Supplementary Fig. S9). In summary, we demonstrated that NOTCH1 positively regulates HER3 through transcriptional control and increased deubiquitination and subsequent stabilization of the protein. Loss of NOTCH1 would activate the AKT pathway and suppress the deubiquitination of HER3, in turn the degradation of HER3 will be enhanced (Fig. 5).

## Discussion

HER3 is a member of the HER family, and its primary ligand is NRG1. Although HER3 has weak kinase activity compared to other HER coreceptors, it can interact with EGFR to form kinase-active hetero-oligomers upon binding of the cognate ligands to the extracellular receptor domains [22]. HER3 serves as an efficient phosphotyrosine scaffold upon transphosphorylation by other HER family members and potently activates downstream signaling. Moreover, the cytoplasmic domain of HER3 possesses high specificity for the p85 regulatory subunit of PI3K (six docking sites per HER3 molecule) [23] and HER3 plays a role as pivotal linker between EGFR and PI3K/AKT signaling pathways. While HER3 amplification and/or overexpression is known to occur, only sporadic somatic HER3 mutations have been reported [12, 24, 25]. Protein-altering mutations of HER3 were found only in 1% (1 out of 74 cases) of human head and neck cancers [12]. On the other hand, previous studies have uncovered coexpression of HER3 and NRG1, and subsequent autocrine signaling, in HNSCC cell lines and clinical samples [26]. Moreover, high NRG1 expression was reported to be associated with activated HER3 in HNSCC and was proposed as a selective biomarker [27]. However, no detailed information about the spatial distribution of the receptor and ligand were given in previous studies. In our study, we found that HER3- and NRG1-positive cells were prevalent in SCCHN from Taiwanese patients, but a significant correlation with undesirable clinical features was only found for tumors with concomitant high levels of HER3 and NRG1. Moreover, the majority of HER3 was observed in differentiated squamous cells at the center of infiltrative tumor nests, while the NRG1 protein was mainly detected in the basaloid cells at the periphery of tumor islands. These observations prompted us to explore whether HER3 expression is under the regulation of differentiation pathways in the squamous epithelium.

NOTCH1 is a master regulator of terminal differentiation in the stratified squamous epithelium, and its expression is mainly present on the differentiated keratinocytes of the spinous layer [28, 29]. Once engaged by various ligands of the Delta-like and Jagged protein families, two proteolytic cleavages (first by ADAM10 and then by γ-secretase) take place on NOTCH1, releasing the Notch intracellular domain (NICD). The NICD subsequently enters the nucleus and stimulates transcription of target genes in cooperation with the DNA-binding protein, CBF1-Suppressor of Hairless-LAG1 (CSL; also known as RBPJ), and the co-activator, Mastermind-like transcriptional co-activator 1 (MAML1) [30–32]. Our study revealed that downregulation of NOTCH1 in SCCHN cells caused decreased expression of HER3, and functional suppression of γ-secretase lead to similar results. In contrast, constitutive overexpression of NOTCH1 increases the expression of HER3. These results were in agreement with the finding that most of the positive immunostaining signal for HER3 was detected in differentiated tumor cells of clinical samples. In addition, our data demonstrated that exposure to NRG1 leads to downregulation of HER3 in NOTCH1-low expressing cells. This finding supports the observation that the peripheral undifferentiated basaloid cells in tumor nests exhibit a low level of HER3 and NOTCH1 and a high level of NRG1. Further experiments showed that loss of NOTCH1 function results in destabilization of HER3 protein through increased phosphorylation of AKT at serine 437 and augmented HER3 polyubiquitination. Polyubiquitination is a highly-conserved eukaryotic system for proteosomal degradation of proteins [33]. After interaction with a ubiquitin-activating enzyme (E1), ubiquitin is transferred to a ubiquitin-conjugating enzyme (E2) and forms polyubiquitin chains. The polyubiquitin chain will be attached to a target protein through a ubiquitin-protein ligase (E3). The poly-ubiquitinated target protein is then usually destined to be recognized and degraded by the 26 S proteasome. Interestingly, our data revealed that the expression of two major E3 ligases, NEDD4 and NRDP1, was not affected by the knockdown of NOTCH1. Yet ubiquitin modifications are reversible, as the conjugates can be removed from substrates by the action of deubiquitinating enzymes (DUBs) [34]. The largest DUB family is the ubiquitin-specific proteases; the members of this family are essential regulators of a wide range of different cellular functions. For instance, USP7 controls p53 stability by deubiquitination of p53 and Mdm2 [35], and USP1 is known to regulate the DNA replication processivity factor, PCNA, by cleaving the ubiquitinated protein [36]. Among USPs, USP8 is a negatively regulated substrate of the AKT pathway, as PTEN loss and AKT activation are associated with suppression of USP8 levels [37]. Previous studies showed that USP8 participates in the endosomal sorting of transmembrane proteins [38, 39] and modulates their function and stability through deubiquitination. Furthermore, USP8 was found to promote degradation of EGFR [38, 40] and hepatocyte growth factor receptor [38]. In the current study, we demonstrated that the polyubiquitination and subsequent reduction of HER3 is enhanced by USP8 attenuation via activation of the AKT pathway in SCCHN cells with loss of NOTCH1 function.

Although NOTCH1 is considered to be oncogenic in several types of malignancies, studies utilizing whole-exome sequencing detected inactivating mutations of NOTCH1 in 12–15% of SCCHN cases [9, 12]. In addition, increased cutaneous tumorigenesis is evident in animal models with disabled NOTCH1 [41]. These findings suggest that NOTCH1 confers tumor-suppressive functions in the stratified squamous epithelium, where SCCHN arises. However, conflicting reports claiming oncogenic mechanisms conferred by NOTCH1 in SCCHN also exist [42–44]. This raises the possibility that the result of NOTCH1 activation is contextual in SCCHN and is influenced by the crosstalk between other signaling pathways [45]. Our data showed that loss of NOTCH1 function activates the AKT pathway and leads to suppression of HER3 in SCCHN cells. This novel finding is intriguing since HER3 is considered to be a potent activator of PI3K, and it seems to be a paradox that loss of function of a tumor suppressor gene causes downregulation of an oncogenic receptor tyrosine kinase at first glance. Nevertheless, the relative abundance of EGFR and HER3 and the presence of EGF or NRG triggers different oligomeric combinations and the downstream pathways [46], and heterodimerization between EGFR and HER3 occurs only when HER3 is engaged by NRG1 but not when EGFR is bound by EGF. In short, the function of HER3 is determined by the abundance of NRG1, and the combined evaluation of this pair of receptors and ligands is more appropriate in the clinical setting. In addition, activation of AKT can trigger a negative feedback regulation upon HER3, as demonstrated in breast cancers [15]. The exact mechanism of NOTCH1-AKT coupling is currently unclear. NOTCH1-regulated MYC has been shown to be a transcriptional activator of PTEN [47], and downregulation of PTEN leads to increased AKT signaling. In addition, multiple factors influence the outcome of activated NOTCH1 signaling [48]. For instance, the signaling strength of NOTCH1 activation depends on the type of engaged ligand and the relative levels between the ligands and NOTCH1 per se [49]. Glycosylation of EGF repeats in the extracellular domain of NOTCH1 by Fringe protein in Golgi bodies also modulates the signaling pattern of NOTCH1 [50]. These features render immunohistochemical quantification alone against the protein backbone of NOTCH1 as a suboptimal indicator for the activity of the downstream pathway. Other than the canonical pathway, NOTCH1 can also exert biological function through a non-canonical, ligand-independent route involving β-catenin [51]. A comprehensive multi-omic analysis in SCCHN may shed light on finding the missing link between NOTCH1 and AKT, and an animal model with an inducible knockout of NOTCH1 is needed to serve as a more authentic platform for the exploration of the role of NOTCH1 in various stages of precancerous lesions to SCCHN. In summary, our data suggested that NOTCH1 is a higher-ranked regulator of the AKT axis than HER3. As a consequence, our results offer some insight into how strategies and patient stratification may be improved in future clinical trials for anti-HER3 therapeutics in SCCHN. Currently, various HER3-targeting therapeutic monoclonal antibodies have been developed and are entering clinical trials, including patritumab/U3–1287 [52], seribantumab/MM-121 [53], and lumretuzumab/RG7116 [54]. However, none of these HER3-targeting antibodies has been approved for clinical use because of unsatisfactory clinical benefit. Since NOTCH1 is inactivated in approximately one-tenth of SCCHN cases, and this aberration is a powerful activator of the AKT pathway and a negative regulator of HER3, exclusion of patients bearing NOTCH1-inactivated SCCHN tumors may be recommended for future clinical trials on HER3-targeting antibodies. In addition, the development of an antibody-drug conjugate (ADC), with a cytotoxic payload attached to an anti-HER3 antibody, may be a promising strategy, owing to the high prevalence of HER3 expression in SCCHN. Thus, HER3 may serve as a molecular beacon for drug delivery that overcomes compensatory signaling crosstalk in malignant cells. This strategy is currently under investigation [55], and the preclinical results show good antitumor activity against HER3-expressing tumors with tolerable safety profiles. In conclusion, our data suggest that HER3 is under the control of NOTCH1, which stimulates HER3 transcriptional activation and inhibits its protein degradation. Interestingly, loss of function of NOTCH1 suppressed the expression of HER3 but boosted the phosphorylation of AKT serine 473 (S473) in SCCHN cells, suggesting that NOTCH1 affects the PI3K/AKT pathway more profoundly and independently of its effects on HER3.

## Supplementary information

Supplementary information
