# Supplementary material for: NOTCH1 signaling promotes protein stability of HER3 through the AKT pathway in squamous cell carcinoma of head and neck
Source: Oncogenesis. 2021 Aug 31;10(8):59. doi: 10.1038/s41389-021-00348-5 (PMC8408252; doi:10.1038/s41389-021-00348-5)
Supplement: Supplementary file 1 — Supplementary information [file 41389_2021_348_MOESM1_ESM.docx]

**Supplementary Materials and Methods**

***In silico* analysis of NOTCH1 and HER3 mRNA expression using the cancer genome atlas (TCGA) normal tissue database**

The expression status of mRNA of NRG1 was performed through accessing to the database of a TCGA cohort of primary SCCHN (n = 520) and normal tissue (n = 44) on the UALCAN platform (<http://ualcan.path.uab.edu/>)

**Plasmids**

The full-length NOTCH1 plasmid RC211365 (Origene, USA) was used for overexpression of NOTCH1 in target cells. To establish NOTCH1-knockdown stable SCCHN cell lines, lentiviruses expressing short RNA (shRNA) sequences against human NOTCH1 (shN-2, 5'-CCGGGACATCACGGATCATAT-3'; shN-9, 5'-AGGGAAGTTGAACGAGCATAG-3') were purchased from the National RNAi Core Facility (Academia Sinica, Taipei, Taiwan). To knock out NOTCH1 with CRISPR-Cas9, the pLentiCRISPR v2 plasmid (JP9388, GenScript, HK) encoding a NOTCH1 single guide RNA (NOTCH1-KO sgRNA; F: 5’- TGCAGGTCAGTACTGTACCG -3’) was introduced into cells by lentivirus infection. At 24 h post-infection, puromycin selection was applied at a concentration of 2 μg/mL for 72 h. The knockout efficiency was determined by RT-PCR (real-time polymerase chain reaction) and Western blotting.

**Lentivirus packaging and transduction**

To produce lentivirus, HEK293T cells were transfected with packaging plasmid (pCMV-△R8.91), envelope (pMDG), and short-hairpin pLKO-RNAi or NOTCH1-KO plasmids using a PolyJET^TM^ transfection kit (SignaGen, USA). One day later, the culture medium containing PolyJET^TM^ was replaced with fresh DMEM medium containing 1% BSA to improve the yield of viral particles. Virus-containing medium was collected twice (24 h after fresh medium replacement) and filtered through a 0.45-μm PVDF sterile filter to remove cell debris. The filtered fluid was mixed with fresh medium containing polybrene (8 μg/ml), and incubated with target cells for another 48 h. Transduced cells were selected with puromycin (4 μg/ml) for 14 days. The efficiency of knockdown and overexpression were determined by RT-PCR and Western blotting.

**RNA extraction and RT-PCR**

The cells were lysed in TRIzol reagent (Invitrogen, USA), and RNA was harvested form the aqueous phase, precipitated with isopropanol and resuspended in DEPC-H_2_O. Complementary DNAs (cDNAs) were generated through reverse-transcription of RNAs (5 μg) with RevertAid H Minus First Strand cDNA Synthesis Kit (Thermo, USA). For RT-PCR, reactions were performed with LightCycler 480 SYBR Green on the LightCycler 480 II platform (Roche, Switzerland). The primer sets for corresponding target genes were β-actin: forward, TTGTTACAGGAAGTCCCTTGCC, and reverse, ATGCTATCACCTCCCCTGTGTG; NOTCH1: forward, ACCAATACAACCCTCTGCGG, and reverse, GGCCCTGGTAGCTCATCATC; HER3: forward, GCCAATGAGTTCACCAGGAT, and reverse, ACGTGGCCGATTAAGTGTTC. The expression levels of genes were normalized to β-actin in the same sample. Each reaction was performed in triplicate. The 2^−ΔΔCT^ method was utilized to analyze results.

**Western blotting**

Cells were lysed in RIPA buffer (20 mM Tris-HCl, 150 mM NaCl, 1 mM EDTA, 1% NP-40, 1% sodium deoxycholate, 2.5 mM sodium pyrophosphate, pH 7.4) containing protease inhibitors (Roche, Switzerland) and phosphatase inhibitors (Roche, Switzerland). The cell lysates were then quantified by Pierce ^TM^ BCA Protein Assay Kit (Thermo, USA), and the absorbance was measured at 562 nm. Protein samples (40 μg) were separated by electrophoresis, and the proteins were then transferred to 0.45-μM PVDF membrane (Millipore, USA). Membranes were blocked with 3% BSA in Tris buffered saline with tween 20 (TBST_0.1_) for 1h at room temperature, then incubated with primary antibody (listed in the Supplementary Table 2) at 4℃ overnight. After washing with TBST_0.1_, secondary antibody (HRP-conjugated goat anti-mouse IgG or HRP-conjugated goat anti-rabbit IgG) was added at room temperature for 1 h. Luminescence from ECL Western blot detection reagents (Millipore, USA) was detected, and the band intensity was quantified with a UVP BioSpectrum image system and normalized to the intensity of α-tubulin. After normalization, the relative fold-change to control was calculated, and the values are displayed under the bands in immunoblots.

**Cell proliferation assay**

Cells were seeded at 2000 cells per well in a 96-well plate. Proliferation was measured via the WST-1 assay (Takara Bio, Japan) for the indicated times. The absorbance of reduced WST-1 was measured at a wavelength of 440 nm.

**Colony formation**

Cells were evaluated. Cells (3×10^3^) were cultured in 6-well plate with 1 mL media containing 10% FBS cultured for 10 days. After incubation, the cells were fixed with 100% methanol and stained with 0.1% crystal violet for 15 min. All experiments were performed in triplicate.

**Migration assay**

Cells were evaluated by using a 24-Transwell insert with 8-μm pores. The cells (1×10^5^) were suspended in fetal bovine serum (FBS)-free medium and lower chambers were then filled with 1 mL media containing 10% FBS cultured for 24 h. After incubation, the cells remaining in the upper side of the insert were removed, and the cells that had infiltrated the lower side of the insert were fixed with 100% methanol and stained with 0.1% crystal violet for 15 min. The stained intensity were counted. All experiments were performed in triplicate.

**Invasion assay**

Cells were evaluated by using a 24-Transwell insert with 8-μm pores coated with Matrigel. Cells (1×10^5^) were suspended in fetal bovine serum (FBS)-free medium and lower chambers were then filled with 1 mL media containing 10% FBS cultured for 24 h. After incubation, the cells remaining in the upper side of the insert were removed, and the cells that had infiltrated the lower side of the insert were fixed with 100% methanol and stained with 0.1% crystal violet for 15 min. The stained intensity were counted. All experiments were performed in triplicate.

.

**Supplementary Fig. S1**

**Comparison of the of the expression of HER3 and NRG1 in SCCHN. (A)** Immunohistochemical staining of HER3 and NRG1 in 11 cases of normal oral mucosa and 100 cases of OSCC. **(B)** mRNA level of NRG1 in the TCGA database of SCCHN and the normal counterpart. The transcript number of NRG1 is significantly higher in primary SCCHN than in the normal mucosa (*p* < 1e-12).

**
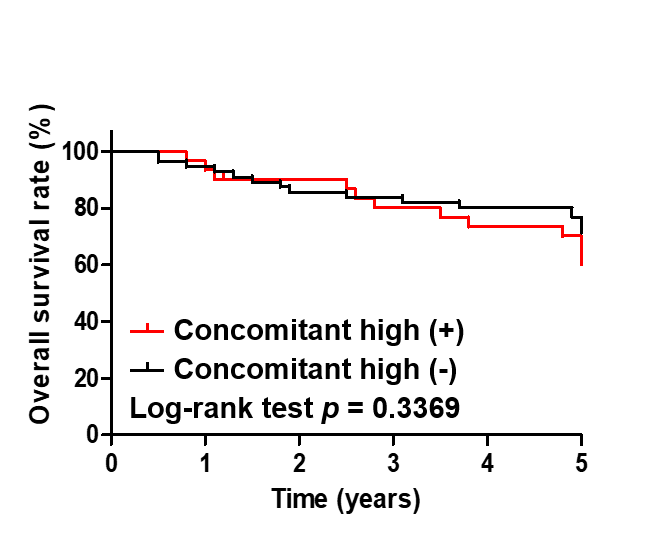
**

**Supplementary Fig. S2**

**Kaplan-Meier overall survival (OS) curves between concomitant NRG1 and HER3 high-expressing subset (n = 31) and the rest of the enrolled cases of OSCC (n = 56).** No significant difference was evident between the two groups.

**
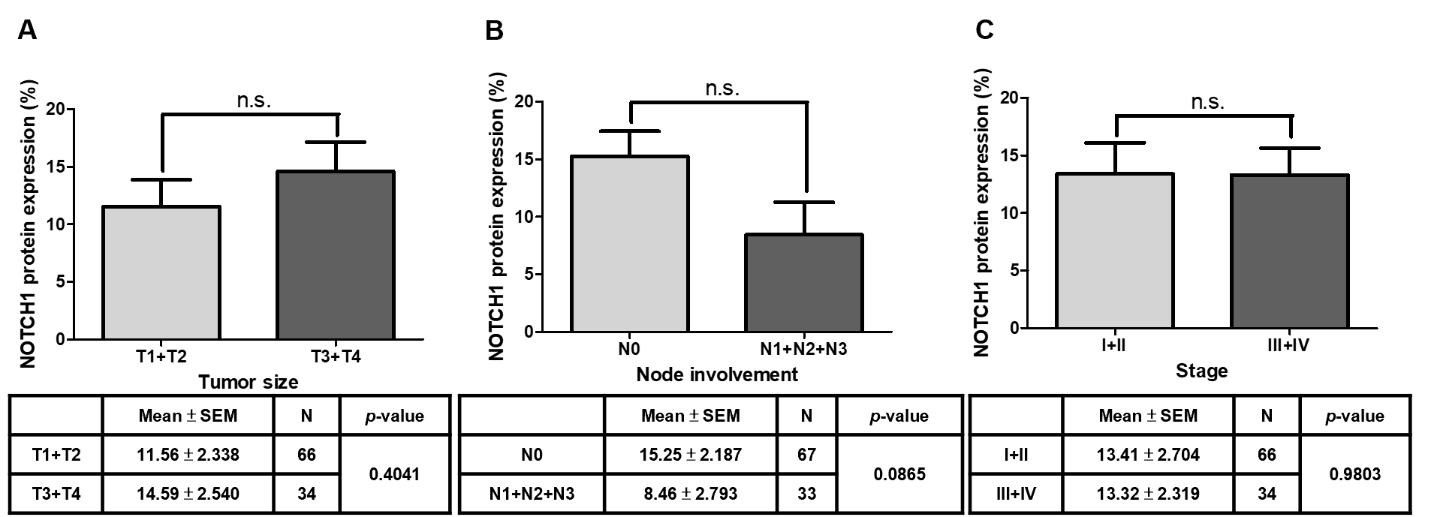
**

**Supplementary Fig. S3**

**Clinicopathological correlation of NOTCH1 and parameters of TNM staging system in OSCC.** The expression of NOTCH1 protein in the cases without nodal involvement was approximately two-fold higher than those cases with nodal diseases, and down-regulation of NOTCH1 was closely associated with nodal involvement (*p* = 0.0865).


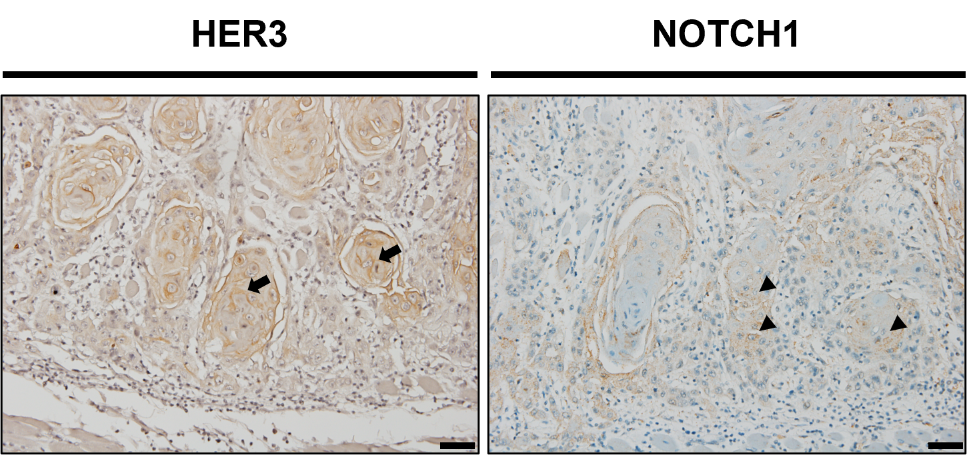


**Supplementary Fig. S4**

**Immunohistochemical staining of HER3 and NOTCH1 in clinical specimens of OSCC.** Colocalization of NOTCH1 and HER3 within the infiltrative tumor nests was discerned in 51% cases of our enrolled cohort of OSCC. The majority of HER3-positive cells were centrally-located differentiated tumor cells in the infiltrating tumor nests (arrow, HER3; arrowhead, NOTCH1).


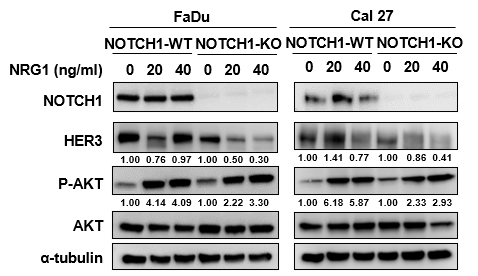


**Supplementary Fig. S5**

**The effect of NRG1 stimulation on HER3 expression in NOTCH1-knockout SCCHN cells.** The expression of HER3 was dose-dependently reduced by treatment of NRG1 in increasing concentrations in NOTCH1-knockout FaDu cells and Cal 27 cells, and this reduction was also present in the NOTCH1-expressing counterparts when exposed to NRG1 in high concentration.


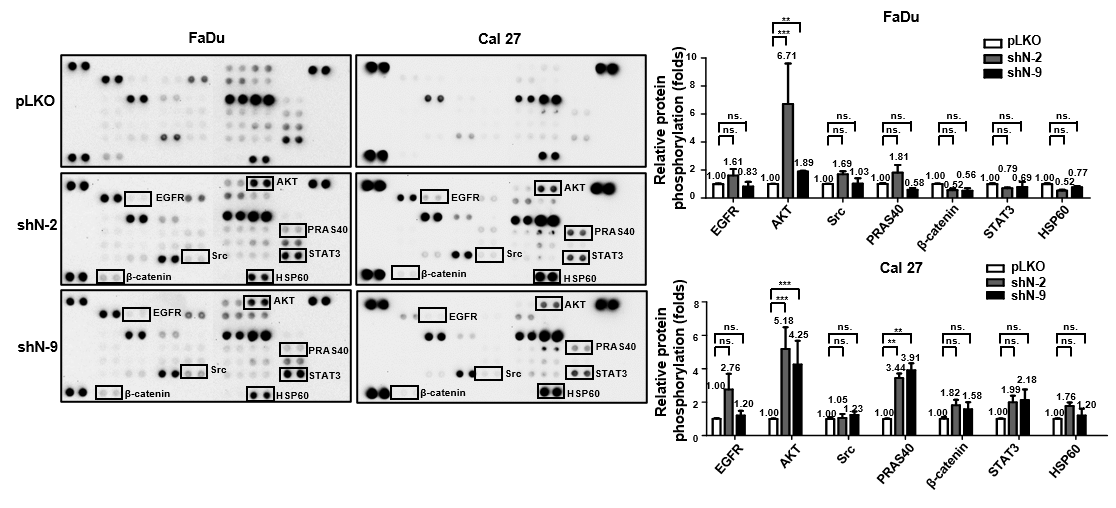


**Supplementary Fig. S6**

**Knockdown of NOTCH1 enhances phosphorylation at serine 473 of AKT in SCCHN cells.** The phosphorylation landscape of receptor tyrosine kinase (RTK) pathways was assessed after knockdown of NOTCH1 in SCCHN cells, using a human Phospho-RTK array. The phosphorylation of AKT serine 473 was increased after NOTCH1 suppression by shRNA in FaDu cells (6.71-fold) **(A)** and Cal 27 cells (5.18-fold) **(B)**. Column: average relative expression normalized to the pLKO group ± standard error (n = 3). **, *p* < 0.01; ***, *p* < 0.001 by two-way ANOVA.


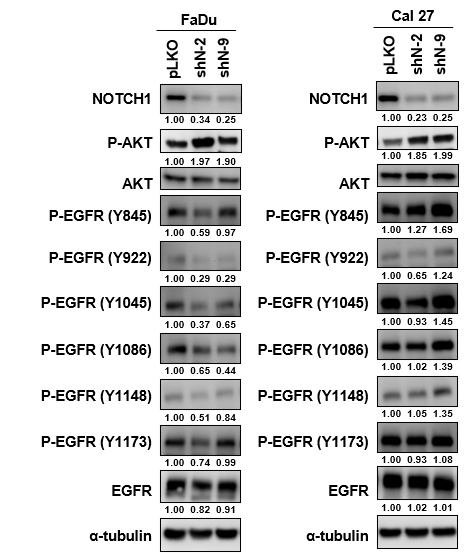


**Supplementary Fig. S7**

**The impact of down-regulation of NOTCH1 on the phosphorylation landscape of EGFR in SCCHN cells.** Although enhanced phosphorylation of AKT was consistently observed, the phosphorylation signatures of EGFR were generally decreased after knockdown of NOTCH1 in FaDu cells, while phosphorylation of EGFR were upregulated by the shN-9 clone in Cal 27 cells. No shared pattern of EGFR phosphorylation after knockdown of NOTCH1 was discerned.

**
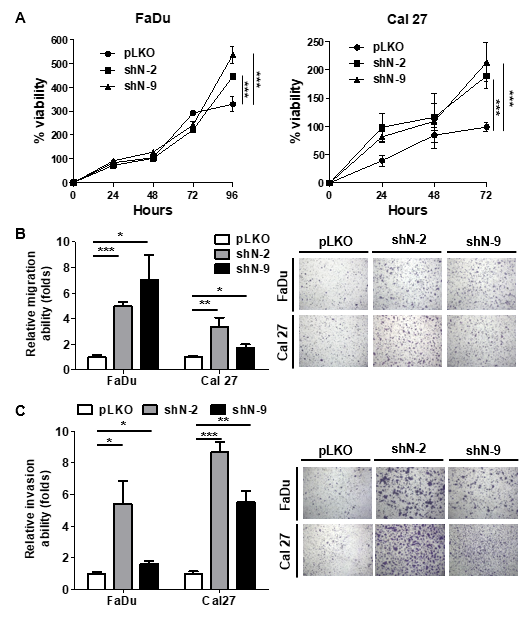
**

**Supplementary Fig. S8**

**Functional assays of the NOTCH1-wild type (pLKO) and NOTCH1-knockdown (shN-2 and shN-9) SCCHN cells.** Knockdown of NOTCH1 with clones of shRNA (shN-2 and shN-9) promotes the cellular viability (A), migration (B), and invasion (C) in both FaDu and Cal 27 cells. However, the effects of shN-9 was less prominently in migration assay of Cal 27 cells and invasion assays in FaDu cells, as compare to those induced by shN-2 shRNA clones (the relative fold changes in migration assay: FaDu-pLKO, 1.00±0.13; FaDu-shN-2, 4.96±0.37; FaDu-shN-9, 7.06±1.94; and Cal 27-pLKO, 1.00±0.71; Cal27-shN-2, 3.35±0.71; Cal 27-shN-9, 1.67±0.32. And the relative fold changes in invasion assay: FaDu-pLKO, 1.00±0.10; FaDu-shN-2, 5.42±1.44; FaDu-shN-9, 1.61±0.13; and Cal 27-pLKO, 1.00±0.13; Cal27-shN-2, 8.67±0.64; Cal 27-shN-9, 5.51±0.71.) Data are presented as the mean ± SD. *, p<0.05; **, p<0.01 and ***, p < 0.001


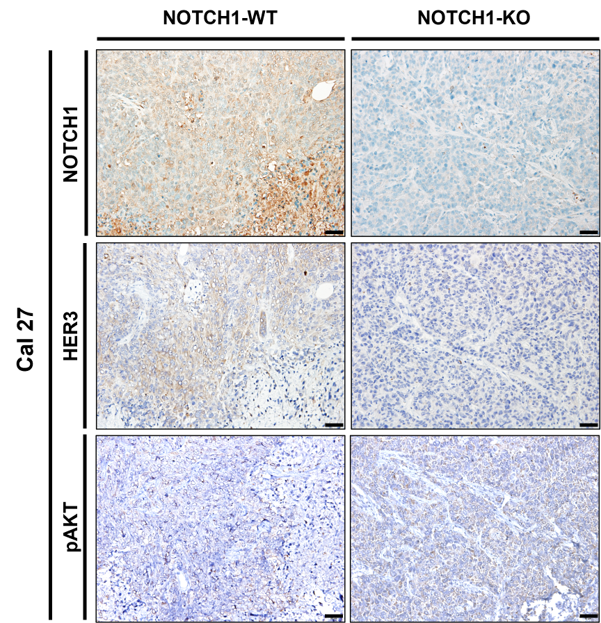


**Supplementary Fig. S9**

**Immunohistochemical staining of HER3, NOTCH1, and phosphorylated AKT in the xenografted Cal 27 cells in the murine subcutaneous xenograft model.** The expression of HER3 was suppressed in NOTCH1-knockout (NOTCH1-KO) cells when compared to the NOTCH1-wild type counterpart, while the phosphorylation of AKT was augmented.

| **Supplementary Table S1.**  **Clinicopathological parameters of OSCC patient cohort** | |
| --- | --- |
| **Parameter** | **Case number** |
| **Gender** |  |
| **Male** | **91** |
| **Female** | **9** |
|  |  |
| **Age** |  |
| **<50 y/o** | **38** |
| **≧50 y/o** | **62** |
|  |  |
| **Differentiation** |  |
| **Well/moderate** | **97** |
| **Poor** | **3** |
|  |  |
| **T** |  |
| **T1+T2** | **42** |
| **T3+T4** | **58** |
|  |  |
| **N** |  |
| **N0** | **72** |
| **N1+N2+N3** | **28** |
|  |  |
| **Stage** |  |
| **I+II** | **34** |
| **III+IV** | **66** |
|  |  |

| **Supplementary Table S2.**  **Primary antibodies** | | | |
| --- | --- | --- | --- |
| **Application** | **Antibody** | **Brand**  **Cat. No. / Clone** | **Dilution** |
| **IHC** | **HER3** | **Cell Signaling Technology**  **#12708 / D22C5** | **1:100** |
|  | **NRG1** | **GeneTex**  **GTX22369 / 7D5** | **1:100** |
|  | **NOTCH1** | **Merck 07-1232** | **1:400** |
|  | **P-AKT** | **Proteintech**  **66444-1-Ig / 1C10B8** | **1:100** |
| **WB** | **α-tubulin** | **Sigma-Aldrich Corporation**  **T5168 / B-5-1-2** | **1:20000** |
|  | **HER3** | **Cell Signaling Technology**  **#12708 / D22C5** | **1:1000** |
|  | **P-HER3**  **(Y1289)** | **Cell Signaling Technology**  **#4791 / 21D3** | **1:1000** |
|  | **NOTCH1** | **Cell Signaling Technology**  **#3608 / D1E11** | **1:1000** |
|  | **cl-NOTCH1** | **Cell Signaling Technology**  **#4147 / D3B8** | **1:1000** |
|  | **AKT** | **Cell Signaling Technology**  **#9272** | **1:1000** |
|  | **P-AKT** | **Cell Signaling Technology**  **#9271** | **1:1000** |
|  | **USP8** | **Cell Signaling Technology**  **#11832 / D18F6** | **1:1000** |
|  | **Ub** | **Santa Cruz**  **sc-8017 / P4D1** | **1:1000** |
|  | **Flag** | **Sigma**  **F3165 / M2** | **1:1000** |
|  | **EGFR** | **Cell Signaling Technology**  **#4267 / D38B1** | **1:1000** |
|  | **P-EGFR**  **(Y845)** | **Cell Signaling Technology**  **#2231** | **1:1000** |
|  | **P-EGFR**  **(Y922)** | **Cell Signaling Technology**  **#2235** | **1:1000** |

| **Supplementary Table S2.**  **Primary antibodies (continued)** | | | |
| --- | --- | --- | --- |
| **Application** | **Antibody** | **Brand**  **Cat. No. / Clone** | **Dilution** |
| **WB** | **P-EGFR**  **(Y1045)** | **Cell Signaling Technology**  **#2237** | **1:1000** |
|  | **P-EGFR**  **(Y1086)** | **Cell Signaling Technology**  **#2220** | **1:1000** |
|  | **P-EGFR**  **(Y1148)** | **Cell Signaling Technology**  **#4404** | **1:1000** |
|  | **P-EGFR**  **(Y1173)** | **Cell Signaling Technology**  **#4407** | **1:1000** |
